# 4-(3-Phenyl-3,3a,4,5-tetra­hydro-2*H*-benzo[*g*]indazol-2-yl)benzene­sulfonamide ethanol monosolvate

**DOI:** 10.1107/S1600536812028474

**Published:** 2012-06-30

**Authors:** Abdullah M. Asiri, Hassan M. Faidallah, Khalid A. Alamry, Seik Weng Ng, Edward R. T. Tiekink

**Affiliations:** a Center of Excellence for Advanced Materials Research (CEAMR), King Abdulaziz University, PO Box 80203, Jeddah 21589, Saudi Arabia; b Chemistry Department, Faculty of Science, King Abdulaziz University, PO Box 80203, Jeddah 21589, Saudi Arabia; c Department of Chemistry, University of Malaya, 50603 Kuala Lumpur, Malaysia

## Abstract

In the title compound ethanol monosolvate, C_23_H_21_N_3_O_2_S·C_2_H_5_OH, the dihydro­pyrazole ring is twisted about the C*sp* ^3^—C*sp* ^3^ bond. Nevertheless, the ring approximates a plane (r.m.s. deviation for the fitted atoms = 0.132 Å) and forms dihedral angles of 5.80 (13) and 12.29 (12)°, respectively, with the fused- and sulfonamide-benzene rings. As the dihydro­pyrazole C-bound phenyl group is roughly perpendicular to the dihydro­pyrazole ring [dihedral angle = 74.04 (15)°; the amino group is orientated to the same side of the mol­ecule], to a first approximation, the mol­ecule has a stunted T-shape. The cyclo­hexene ring adopts a half-chair conformation with the methyl­ene C atom connected to the dihydro­pyrazole ring lying 0.665 (4) Å out of the plane of the five remaining atoms (r.m.s. deviation = 0.050 Å). The components of the asymmetric unit are connected by an O—H⋯O hydrogen bond. Further links between mol­ecules leading to a three-dimensional architecture are of the type N—H⋯O.

## Related literature

For a previous synthesis, see: Faidallah & Makki (1994 ▶). For the biological activity of related compounds, see: Faidallah *et al.* (2011 ▶). For the structure of the methyl analogue, see: Asiri *et al.* (2011 ▶).
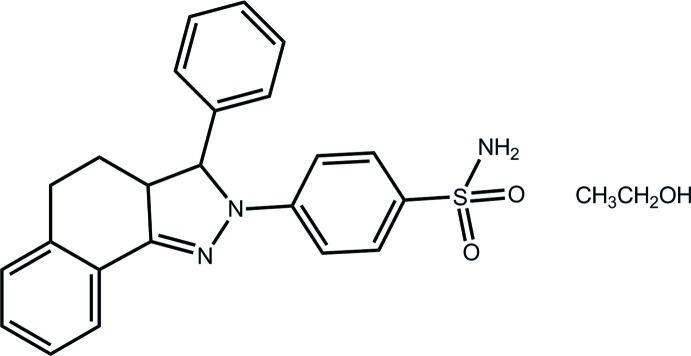


## Experimental

### 

#### Crystal data

C_23_H_21_N_3_O_2_S·C_2_H_6_O
*M* *_r_* = 449.56
Monoclinic, 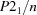

*a* = 15.7556 (9) Å
*b* = 9.1789 (4) Å
*c* = 16.7515 (10) Å
β = 111.718 (7)°
*V* = 2250.6 (2) Å^3^
*Z* = 4
Mo *K*α radiation
μ = 0.18 mm^−1^
*T* = 100 K
0.30 × 0.25 × 0.20 mm

#### Data collection

Agilent SuperNova Dual diffractometer with an Atlas detector
Absorption correction: multi-scan (*CrysAlis PRO*; Agilent, 2012 ▶) *T*_min_ = 0.761, *T*_max_ = 1.000
15054 measured reflections
5196 independent reflections
3971 reflections with *I* > 2σ(*I*)
*R*_int_ = 0.036

#### Refinement

*R*[*F* ^2^ > 2σ(*F* ^2^)] = 0.054
*wR*(*F* ^2^) = 0.156
*S* = 1.04
5196 reflections
301 parameters
3 restraints
H atoms treated by a mixture of independent and constrained refinement
Δρ_max_ = 0.81 e Å^−3^
Δρ_min_ = −0.45 e Å^−3^

### 

Data collection: *CrysAlis PRO* (Agilent, 2012 ▶); cell refinement: *CrysAlis PRO*; data reduction: *CrysAlis PRO*; program(s) used to solve structure: *SHELXS97* (Sheldrick, 2008 ▶); program(s) used to refine structure: *SHELXL97* (Sheldrick, 2008 ▶); molecular graphics: *ORTEP-3 for Windows* (Farrugia, 1997 ▶) and *DIAMOND* (Brandenburg, 2006 ▶); software used to prepare material for publication: *publCIF* (Westrip, 2010 ▶).

## Supplementary Material

Crystal structure: contains datablock(s) global, I. DOI: 10.1107/S1600536812028474/xu5573sup1.cif

Structure factors: contains datablock(s) I. DOI: 10.1107/S1600536812028474/xu5573Isup2.hkl

Supplementary material file. DOI: 10.1107/S1600536812028474/xu5573Isup3.cml

Additional supplementary materials:  crystallographic information; 3D view; checkCIF report

## Figures and Tables

**Table 1:** Hydrogen-bond geometry (Å, °)

*D*—H⋯*A*	*D*—H	H⋯*A*	*D*⋯*A*	*D*—H⋯*A*
O3—H3*o*⋯O1	0.84 (1)	2.04 (1)	2.875 (2)	175 (3)
N3—H1*n*⋯O3^i^	0.87 (1)	2.02 (1)	2.894 (3)	176 (3)
N3—H2*n*⋯O2^ii^	0.88 (1)	2.16 (1)	3.007 (3)	163 (2)

Symmetry codes: (i) 
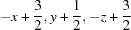
; (ii) 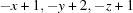.

**Fig. 1.**
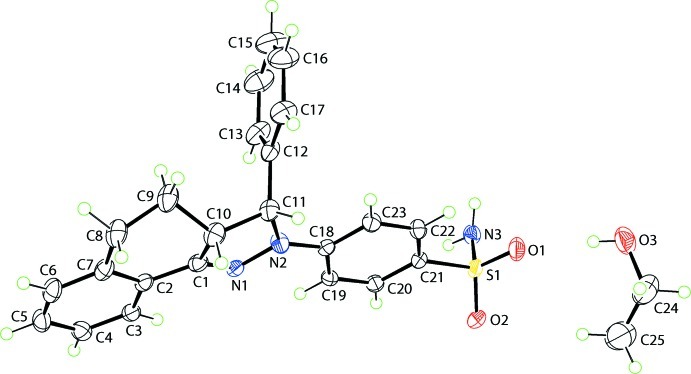
The molecular structure of (I) showing the atom-labelling scheme and displacement ellipsoids at the 50% probability level.

**Fig. 2.**
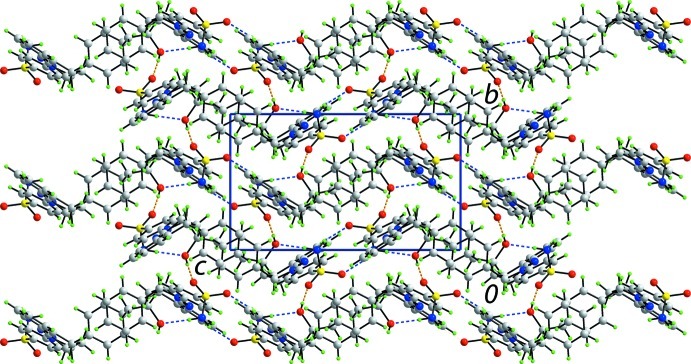
A view in projection down the a axis of the unit-cell contents of (I). The O—H···O and N—H···O hydrogen are shown as orange and blue dashed lines, respectively.

## supplementary crystallographic information

### Comment

The title compound, (I), reported previously in the literature (Faidallah & Makki, 1994), comprises a benzenesulfonamide unit which is grafted to a chemotherapeutic heterocycle pyrazole derivative, and therefore is a compound which is anticipated to exhibit enhanced activities (Faidallah *et al.*, 2011).

In (I), Fig. 1, pyrazole ring is twisted about the C10—C11 bond (r.m.s. deviation for the fitted atoms = 0.132 Å). The cyclohexene ring adopts a half-chair conformation with the C9 atom lying 0.665 (4) Å out of the plane of the five remaining atoms (r.m.s. deviation = 0.050 Å). The fused-ring- and sulfonamide-benzene rings form dihedral angles of 5.80 (13) and 12.29 (12)°, respectively, with the least-squares plane through the pyrazole ring. By contrast, the pyrazole-C-bound phenyl group is almost perpendicular to the pyrazole ring, forming a dihedral angle of 74.04 (15)°, so that to a first approximation, the molecule has a stunted T-shape. The sulfonamide-amino group is orientated to the same side of the molecule as the pyrazole-C-bound benzene ring. While the sulfonamide-O1 atom is almost co-planar with the benzene ring, the O1—S1—C21—C20 torsion angle is -168.51 (17)°, the O2 atom is somewhat splayed [O2—S1—C21—C20 = -38.9 (2)°]. In the structure of the compound where the pyrazole-C-bound substituent is methyl rather than phenyl, the molecule has a shallow bowl-shaped conformation (Asiri *et al.*, 2011).

The asymmetric unit comprises the organic molecule and a ethanol molecule of solvation with the primary connection between them being a O—H···O hydrogen bond, Table 1. Each amino-H forms a hydrogen bond to an oxygen atom so that each oxygen atom in the structure functions as an acceptor, Table 1, and that a three-dimensional architecture results, Fig. 2.

### Experimental

A solution of 2-benzylidene-3,4-dihydro-2*H*-naphthalen-1-one (2.3 g, 0.01 *M*) in ethanol (50 ml) was refluxed with 4-hydrazinobenzenesulfonamide hydrochloride (2.2 g, 0.01 *M*) for 4 h. The reaction mixture was allowed to cool. The formed precipitate was filtered, washed with water, dried and recrystallized from ethanol. *M*.pt: 508–510 K *cf*. Lit. *M*.pt: 508 K (Faidallah & Makki, 1994). Yield: 70%.

### Refinement

Carbon-bound H-atoms were placed in calculated positions [C—H = 0.95–1.00 Å, *U*_iso_(H) = 1.2–1.5*U*_eq_(C)] and were included in the refinement in the riding model approximation. The oxygen- and nitrogen-bound H-atom were located in a difference Fourier map and was refined with a distance restraints of O—H = 0.84±0.01 Å and N—H = 0.88±0.01 Å; the *U*_iso_ values were refined.

### Crystal data

**Table:**

C_23_H_21_N_3_O_2_S·C_2_H_6_O	*F*(000) = 952
*M**_r_* = 449.56	*D*_x_ = 1.327 Mg m^−^^3^
Monoclinic, *P*2_1_/*n*	Mo *K*α radiation, λ = 0.71073 Å
Hall symbol: -P 2yn	Cell parameters from 4495 reflections
*a* = 15.7556 (9) Å	θ = 2.5–27.5°
*b* = 9.1789 (4) Å	µ = 0.18 mm^−^^1^
*c* = 16.7515 (10) Å	*T* = 100 K
β = 111.718 (7)°	Prsim, light-brown
*V* = 2250.6 (2) Å^3^	0.30 × 0.25 × 0.20 mm
*Z* = 4	

### Data collection

**Table:**

Agilent SuperNova Dual diffractometer with an Atlas detector	5196 independent reflections
Radiation source: SuperNova (Mo) X-ray Source	3971 reflections with *I* > 2σ(*I*)
Mirror monochromator	*R*_int_ = 0.036
Detector resolution: 10.4041 pixels mm^-1^	θ_max_ = 27.6°, θ_min_ = 2.6°
ω scan	*h* = −20→16
Absorption correction: multi-scan (*CrysAlis PRO*; Agilent, 2012)	*k* = −11→11
*T*_min_ = 0.761, *T*_max_ = 1.000	*l* = −17→21
15054 measured reflections	

### Refinement

**Table:**

Refinement on *F*^2^	Primary atom site location: structure-invariant direct methods
Least-squares matrix: full	Secondary atom site location: difference Fourier map
*R*[*F*^2^ > 2σ(*F*^2^)] = 0.054	Hydrogen site location: inferred from neighbouring sites
*wR*(*F*^2^) = 0.156	H atoms treated by a mixture of independent and constrained refinement
*S* = 1.04	*w* = 1/[σ^2^(*F*_o_^2^) + (0.0701*P*)^2^ + 1.7118*P*] where *P* = (*F*_o_^2^ + 2*F*_c_^2^)/3
5196 reflections	(Δ/σ)_max_ = 0.001
301 parameters	Δρ_max_ = 0.81 e Å^−^^3^
3 restraints	Δρ_min_ = −0.45 e Å^−^^3^

### Special details

**Table:**

Geometry. All e.s.d.'s (except the e.s.d. in the dihedral angle between two l.s. planes) are estimated using the full covariance matrix. The cell e.s.d.'s are taken into account individually in the estimation of e.s.d.'s in distances, angles and torsion angles; correlations between e.s.d.'s in cell parameters are only used when they are defined by crystal symmetry. An approximate (isotropic) treatment of cell e.s.d.'s is used for estimating e.s.d.'s involving l.s. planes.
Refinement. Refinement of *F*^2^ against ALL reflections. The weighted *R*-factor *wR* and goodness of fit *S* are based on *F*^2^, conventional *R*-factors *R* are based on *F*, with *F* set to zero for negative *F*^2^. The threshold expression of *F*^2^ > σ(*F*^2^) is used only for calculating *R*-factors(gt) *etc*. and is not relevant to the choice of reflections for refinement. *R*-factors based on *F*^2^ are statistically about twice as large as those based on *F*, and *R*- factors based on ALL data will be even larger.

### Fractional atomic coordinates and isotropic or equivalent isotropic displacement parameters (Å^2^)

**Table:**

	*x*	*y*	*z*	*U*_iso_*/*U*_eq_	
S1	0.56232 (4)	0.84182 (6)	0.60754 (4)	0.02401 (16)	
O1	0.62832 (11)	0.73972 (19)	0.66013 (11)	0.0328 (4)	
O2	0.52421 (11)	0.82084 (18)	0.51577 (10)	0.0295 (4)	
O3	0.78024 (13)	0.5436 (2)	0.69624 (13)	0.0455 (5)	
N1	0.17020 (12)	0.91470 (19)	0.67783 (11)	0.0199 (4)	
N2	0.25282 (13)	0.8494 (2)	0.72722 (12)	0.0251 (4)	
N3	0.61120 (13)	0.9993 (2)	0.62362 (14)	0.0275 (4)	
C1	0.11176 (15)	0.8807 (3)	0.71164 (14)	0.0243 (5)	
C2	0.01730 (15)	0.9315 (2)	0.68075 (14)	0.0220 (5)	
C3	−0.02015 (15)	1.0140 (2)	0.60582 (14)	0.0235 (5)	
H3	0.0168	1.0402	0.5743	0.028*	
C4	−0.10982 (16)	1.0577 (3)	0.57728 (16)	0.0283 (5)	
H4	−0.1348	1.1142	0.5264	0.034*	
C5	−0.16410 (16)	1.0184 (3)	0.62350 (17)	0.0315 (5)	
H5	−0.2264	1.0472	0.6037	0.038*	
C6	−0.12745 (17)	0.9380 (3)	0.69772 (17)	0.0310 (5)	
H6	−0.1652	0.9116	0.7284	0.037*	
C7	−0.03598 (16)	0.8944 (3)	0.72900 (16)	0.0272 (5)	
C8	0.00372 (18)	0.8092 (3)	0.81182 (17)	0.0358 (6)	
H8A	−0.0152	0.7060	0.8002	0.043*	
H8B	−0.0223	0.8472	0.8532	0.043*	
C9	0.10743 (18)	0.8158 (3)	0.85304 (17)	0.0360 (6)	
H9A	0.1270	0.9154	0.8749	0.043*	
H9B	0.1295	0.7473	0.9021	0.043*	
C10	0.14749 (17)	0.7754 (3)	0.78660 (16)	0.0302 (5)	
H10	0.1262	0.6752	0.7650	0.036*	
C11	0.25155 (16)	0.7839 (3)	0.80769 (15)	0.0262 (5)	
H11	0.2768	0.6828	0.8135	0.031*	
C12	0.30768 (16)	0.8718 (2)	0.88630 (15)	0.0263 (5)	
C13	0.3217 (2)	1.0205 (3)	0.88187 (17)	0.0360 (6)	
H13	0.2957	1.0694	0.8283	0.043*	
C14	0.3723 (2)	1.0976 (3)	0.95360 (19)	0.0465 (7)	
H14	0.3820	1.1990	0.9492	0.056*	
C15	0.4096 (2)	1.0290 (3)	1.0325 (2)	0.0509 (8)	
H15	0.4447	1.0828	1.0822	0.061*	
C16	0.3952 (2)	0.8806 (3)	1.03832 (19)	0.0480 (7)	
H16	0.4194	0.8330	1.0924	0.058*	
C17	0.34566 (19)	0.8023 (3)	0.96556 (17)	0.0363 (6)	
H17	0.3375	0.7004	0.9696	0.044*	
C18	0.32543 (14)	0.8521 (2)	0.70087 (14)	0.0192 (4)	
C19	0.31803 (14)	0.9196 (2)	0.62303 (14)	0.0208 (4)	
H19	0.2631	0.9679	0.5895	0.025*	
C20	0.39002 (15)	0.9158 (2)	0.59542 (14)	0.0223 (5)	
H20	0.3844	0.9610	0.5427	0.027*	
C21	0.47139 (14)	0.8456 (2)	0.64448 (14)	0.0210 (4)	
C22	0.48012 (15)	0.7814 (2)	0.72190 (15)	0.0236 (5)	
H22	0.5356	0.7347	0.7556	0.028*	
C23	0.40790 (15)	0.7853 (2)	0.75024 (14)	0.0236 (5)	
H23	0.4145	0.7420	0.8037	0.028*	
C24	0.7520 (3)	0.4101 (4)	0.6419 (2)	0.0558 (8)	
H24A	0.8069	0.3642	0.6373	0.067*	
H24B	0.7250	0.3397	0.6706	0.067*	
C25	0.6861 (3)	0.4394 (4)	0.5558 (3)	0.0666 (10)	
H25A	0.6701	0.3481	0.5234	0.100*	
H25B	0.7128	0.5071	0.5264	0.100*	
H25C	0.6309	0.4829	0.5597	0.100*	
H1n	0.6416 (19)	1.014 (4)	0.6783 (8)	0.054 (10)*	
H2n	0.5739 (14)	1.066 (2)	0.5919 (14)	0.028 (7)*	
H3o	0.7383 (16)	0.605 (3)	0.687 (2)	0.054 (10)*	

### Atomic displacement parameters (Å^2^)

**Table:**

	*U*^11^	*U*^22^	*U*^33^	*U*^12^	*U*^13^	*U*^23^
S1	0.0220 (3)	0.0289 (3)	0.0241 (3)	0.0055 (2)	0.0119 (2)	−0.0008 (2)
O1	0.0288 (9)	0.0381 (9)	0.0339 (10)	0.0136 (7)	0.0143 (8)	0.0045 (8)
O2	0.0310 (9)	0.0369 (9)	0.0238 (9)	0.0034 (7)	0.0139 (7)	−0.0061 (7)
O3	0.0360 (10)	0.0508 (12)	0.0452 (12)	0.0167 (9)	0.0099 (9)	0.0101 (10)
N1	0.0223 (9)	0.0203 (9)	0.0189 (9)	−0.0011 (7)	0.0099 (8)	−0.0027 (7)
N2	0.0226 (9)	0.0339 (10)	0.0204 (10)	0.0037 (8)	0.0099 (8)	0.0090 (8)
N3	0.0221 (10)	0.0343 (11)	0.0283 (12)	0.0009 (9)	0.0119 (9)	0.0002 (9)
C1	0.0266 (11)	0.0293 (11)	0.0203 (11)	−0.0045 (9)	0.0125 (9)	−0.0027 (9)
C2	0.0235 (11)	0.0227 (10)	0.0236 (11)	−0.0061 (9)	0.0131 (9)	−0.0055 (9)
C3	0.0234 (10)	0.0259 (11)	0.0237 (12)	−0.0062 (9)	0.0115 (9)	−0.0047 (9)
C4	0.0258 (11)	0.0299 (12)	0.0283 (13)	−0.0035 (10)	0.0088 (10)	−0.0022 (10)
C5	0.0230 (11)	0.0333 (12)	0.0415 (15)	−0.0031 (10)	0.0155 (11)	−0.0056 (11)
C6	0.0302 (12)	0.0310 (12)	0.0409 (15)	−0.0044 (10)	0.0236 (12)	−0.0030 (11)
C7	0.0313 (12)	0.0257 (11)	0.0318 (13)	−0.0033 (10)	0.0201 (11)	−0.0033 (10)
C8	0.0378 (14)	0.0435 (14)	0.0380 (15)	0.0015 (12)	0.0276 (13)	0.0073 (12)
C9	0.0399 (14)	0.0433 (14)	0.0336 (14)	0.0112 (12)	0.0239 (12)	0.0132 (12)
C10	0.0352 (13)	0.0294 (12)	0.0313 (13)	0.0011 (10)	0.0187 (11)	0.0038 (10)
C11	0.0290 (12)	0.0305 (11)	0.0229 (12)	0.0030 (10)	0.0141 (10)	0.0065 (9)
C12	0.0333 (12)	0.0255 (11)	0.0243 (12)	0.0089 (10)	0.0158 (10)	0.0044 (9)
C13	0.0557 (17)	0.0276 (12)	0.0300 (14)	0.0099 (12)	0.0220 (13)	0.0045 (10)
C14	0.071 (2)	0.0300 (13)	0.0424 (17)	−0.0030 (14)	0.0262 (16)	−0.0046 (12)
C15	0.068 (2)	0.0465 (17)	0.0341 (16)	−0.0026 (15)	0.0144 (15)	−0.0128 (13)
C16	0.064 (2)	0.0488 (17)	0.0250 (14)	0.0069 (15)	0.0094 (14)	0.0021 (12)
C17	0.0504 (16)	0.0290 (12)	0.0283 (13)	0.0059 (12)	0.0132 (12)	0.0062 (10)
C18	0.0219 (10)	0.0179 (10)	0.0195 (11)	−0.0021 (8)	0.0095 (9)	−0.0024 (8)
C19	0.0193 (10)	0.0249 (11)	0.0171 (11)	0.0006 (8)	0.0056 (9)	0.0003 (8)
C20	0.0234 (11)	0.0264 (11)	0.0182 (11)	0.0006 (9)	0.0088 (9)	0.0012 (9)
C21	0.0207 (10)	0.0217 (10)	0.0215 (11)	0.0011 (8)	0.0090 (9)	−0.0030 (8)
C22	0.0228 (10)	0.0220 (10)	0.0256 (12)	0.0038 (9)	0.0083 (9)	0.0023 (9)
C23	0.0265 (11)	0.0246 (11)	0.0204 (11)	0.0040 (9)	0.0095 (9)	0.0048 (9)
C24	0.071 (2)	0.0537 (19)	0.052 (2)	0.0154 (17)	0.0332 (18)	−0.0003 (15)
C25	0.067 (2)	0.071 (2)	0.064 (3)	−0.0048 (19)	0.027 (2)	−0.0164 (19)

### Geometric parameters (Å, º)

**Table:**

S1—O1	1.4345 (17)	C10—C11	1.546 (3)
S1—O2	1.4414 (17)	C10—H10	1.0000
S1—N3	1.613 (2)	C11—C12	1.518 (3)
S1—C21	1.759 (2)	C11—H11	1.0000
O3—C24	1.493 (4)	C12—C17	1.393 (3)
O3—H3o	0.839 (10)	C12—C13	1.389 (3)
N1—C1	1.285 (3)	C13—C14	1.368 (4)
N1—N2	1.393 (3)	C13—H13	0.9500
N2—C18	1.370 (3)	C14—C15	1.384 (4)
N2—C11	1.483 (3)	C14—H14	0.9500
N3—H1n	0.873 (10)	C15—C16	1.390 (4)
N3—H2n	0.877 (10)	C15—H15	0.9500
C1—C2	1.459 (3)	C16—C17	1.381 (4)
C1—C10	1.518 (3)	C16—H16	0.9500
C2—C3	1.396 (3)	C17—H17	0.9500
C2—C7	1.406 (3)	C18—C23	1.398 (3)
C3—C4	1.373 (3)	C18—C19	1.409 (3)
C3—H3	0.9500	C19—C20	1.375 (3)
C4—C5	1.397 (3)	C19—H19	0.9500
C4—H4	0.9500	C20—C21	1.398 (3)
C5—C6	1.375 (4)	C20—H20	0.9500
C5—H5	0.9500	C21—C22	1.384 (3)
C6—C7	1.398 (3)	C22—C23	1.386 (3)
C6—H6	0.9500	C22—H22	0.9500
C7—C8	1.512 (3)	C23—H23	0.9500
C8—C9	1.522 (4)	C24—C25	1.457 (5)
C8—H8A	0.9900	C24—H24A	0.9900
C8—H8B	0.9900	C24—H24B	0.9900
C9—C10	1.515 (3)	C25—H25A	0.9800
C9—H9A	0.9900	C25—H25B	0.9800
C9—H9B	0.9900	C25—H25C	0.9800
			
O1—S1—O2	119.27 (10)	N2—C11—C10	100.52 (18)
O1—S1—N3	106.86 (11)	C12—C11—C10	116.95 (19)
O2—S1—N3	106.42 (11)	N2—C11—H11	109.0
O1—S1—C21	107.25 (10)	C12—C11—H11	109.0
O2—S1—C21	107.81 (10)	C10—C11—H11	109.0
N3—S1—C21	108.93 (10)	C17—C12—C13	118.7 (2)
C24—O3—H3o	114 (2)	C17—C12—C11	119.3 (2)
C1—N1—N2	107.35 (18)	C13—C12—C11	121.9 (2)
C18—N2—N1	120.57 (17)	C14—C13—C12	120.9 (2)
C18—N2—C11	126.45 (18)	C14—C13—H13	119.5
N1—N2—C11	112.97 (16)	C12—C13—H13	119.5
S1—N3—H1n	111 (2)	C13—C14—C15	120.5 (3)
S1—N3—H2n	110.5 (17)	C13—C14—H14	119.8
H1n—N3—H2n	121 (3)	C15—C14—H14	119.8
N1—C1—C2	124.7 (2)	C14—C15—C16	119.4 (3)
N1—C1—C10	114.3 (2)	C14—C15—H15	120.3
C2—C1—C10	120.91 (19)	C16—C15—H15	120.3
C3—C2—C7	120.3 (2)	C17—C16—C15	120.1 (3)
C3—C2—C1	121.89 (19)	C17—C16—H16	119.9
C7—C2—C1	117.8 (2)	C15—C16—H16	119.9
C4—C3—C2	120.6 (2)	C16—C17—C12	120.4 (3)
C4—C3—H3	119.7	C16—C17—H17	119.8
C2—C3—H3	119.7	C12—C17—H17	119.8
C3—C4—C5	119.6 (2)	N2—C18—C23	120.35 (19)
C3—C4—H4	120.2	N2—C18—C19	120.88 (19)
C5—C4—H4	120.2	C23—C18—C19	118.76 (19)
C6—C5—C4	120.1 (2)	C20—C19—C18	120.3 (2)
C6—C5—H5	120.0	C20—C19—H19	119.9
C4—C5—H5	120.0	C18—C19—H19	119.9
C5—C6—C7	121.5 (2)	C19—C20—C21	120.4 (2)
C5—C6—H6	119.3	C19—C20—H20	119.8
C7—C6—H6	119.3	C21—C20—H20	119.8
C6—C7—C2	117.9 (2)	C22—C21—C20	119.90 (19)
C6—C7—C8	120.8 (2)	C22—C21—S1	120.66 (17)
C2—C7—C8	121.4 (2)	C20—C21—S1	119.44 (16)
C7—C8—C9	113.95 (19)	C21—C22—C23	120.0 (2)
C7—C8—H8A	108.8	C21—C22—H22	120.0
C9—C8—H8A	108.8	C23—C22—H22	120.0
C7—C8—H8B	108.8	C22—C23—C18	120.6 (2)
C9—C8—H8B	108.8	C22—C23—H23	119.7
H8A—C8—H8B	107.7	C18—C23—H23	119.7
C10—C9—C8	109.0 (2)	C25—C24—O3	113.2 (3)
C10—C9—H9A	109.9	C25—C24—H24A	108.9
C8—C9—H9A	109.9	O3—C24—H24A	108.9
C10—C9—H9B	109.9	C25—C24—H24B	108.9
C8—C9—H9B	109.9	O3—C24—H24B	108.9
H9A—C9—H9B	108.3	H24A—C24—H24B	107.7
C9—C10—C1	108.90 (19)	C24—C25—H25A	109.5
C9—C10—C11	121.0 (2)	C24—C25—H25B	109.5
C1—C10—C11	101.19 (17)	H25A—C25—H25B	109.5
C9—C10—H10	108.4	C24—C25—H25C	109.5
C1—C10—H10	108.4	H25A—C25—H25C	109.5
C11—C10—H10	108.4	H25B—C25—H25C	109.5
N2—C11—C12	111.94 (19)		
			
C1—N1—N2—C18	172.3 (2)	C9—C10—C11—C12	−16.4 (3)
C1—N1—N2—C11	−8.9 (2)	C1—C10—C11—C12	103.9 (2)
N2—N1—C1—C2	177.9 (2)	N2—C11—C12—C17	−153.5 (2)
N2—N1—C1—C10	−4.5 (3)	C10—C11—C12—C17	91.3 (3)
N1—C1—C2—C3	5.3 (3)	N2—C11—C12—C13	27.2 (3)
C10—C1—C2—C3	−172.2 (2)	C10—C11—C12—C13	−88.0 (3)
N1—C1—C2—C7	−174.8 (2)	C17—C12—C13—C14	0.5 (4)
C10—C1—C2—C7	7.8 (3)	C11—C12—C13—C14	179.8 (2)
C7—C2—C3—C4	−1.3 (3)	C12—C13—C14—C15	−1.0 (4)
C1—C2—C3—C4	178.6 (2)	C13—C14—C15—C16	0.1 (5)
C2—C3—C4—C5	−0.3 (3)	C14—C15—C16—C17	1.3 (5)
C3—C4—C5—C6	0.8 (4)	C15—C16—C17—C12	−1.8 (5)
C4—C5—C6—C7	0.3 (4)	C13—C12—C17—C16	0.9 (4)
C5—C6—C7—C2	−1.9 (4)	C11—C12—C17—C16	−178.5 (2)
C5—C6—C7—C8	178.5 (2)	N1—N2—C18—C23	−179.40 (19)
C3—C2—C7—C6	2.3 (3)	C11—N2—C18—C23	2.0 (3)
C1—C2—C7—C6	−177.6 (2)	N1—N2—C18—C19	−0.6 (3)
C3—C2—C7—C8	−178.0 (2)	C11—N2—C18—C19	−179.3 (2)
C1—C2—C7—C8	2.1 (3)	N2—C18—C19—C20	−177.0 (2)
C6—C7—C8—C9	−159.6 (2)	C23—C18—C19—C20	1.8 (3)
C2—C7—C8—C9	20.8 (3)	C18—C19—C20—C21	−0.3 (3)
C7—C8—C9—C10	−51.6 (3)	C19—C20—C21—C22	−0.9 (3)
C8—C9—C10—C1	58.8 (3)	C19—C20—C21—S1	179.81 (17)
C8—C9—C10—C11	175.3 (2)	O1—S1—C21—C22	12.3 (2)
N1—C1—C10—C9	143.4 (2)	O2—S1—C21—C22	141.86 (18)
C2—C1—C10—C9	−38.9 (3)	N3—S1—C21—C22	−103.05 (19)
N1—C1—C10—C11	14.9 (3)	O1—S1—C21—C20	−168.51 (17)
C2—C1—C10—C11	−167.4 (2)	O2—S1—C21—C20	−38.9 (2)
C18—N2—C11—C12	71.2 (3)	N3—S1—C21—C20	76.2 (2)
N1—N2—C11—C12	−107.5 (2)	C20—C21—C22—C23	0.7 (3)
C18—N2—C11—C10	−163.9 (2)	S1—C21—C22—C23	179.98 (17)
N1—N2—C11—C10	17.4 (2)	C21—C22—C23—C18	0.7 (3)
C9—C10—C11—N2	−137.8 (2)	N2—C18—C23—C22	176.8 (2)
C1—C10—C11—N2	−17.5 (2)	C19—C18—C23—C22	−2.0 (3)

### Hydrogen-bond geometry (Å, º)

**Table:**

*D*—H···*A*	*D*—H	H···*A*	*D*···*A*	*D*—H···*A*
O3—H3*o*···O1	0.84 (1)	2.04 (1)	2.875 (2)	175 (3)
N3—H1*n*···O3^i^	0.87 (1)	2.02 (1)	2.894 (3)	176 (3)
N3—H2*n*···O2^ii^	0.88 (1)	2.16 (1)	3.007 (3)	163 (2)

Symmetry codes: (i) −*x*+3/2, *y*+1/2, −*z*+3/2; (ii) −*x*+1, −*y*+2, −*z*+1.

## References

[bb1] Agilent (2012). *CrysAlis PRO* Agilent Technologies, Yarnton, England.

[bb2] Asiri, A. M., Faidallah, H. M., Al-Youbi, A. O., Makki, M. S. I. T. & Ng, S. W. (2011). *Acta Cryst.* E**67**, o2441. 10.1107/S1600536811033186 PMC3200647 22065029

[bb3] Brandenburg, K. (2006). *DIAMOND* Crystal Impact GbR, Bonn, Germany.

[bb4] Faidallah, H. M., Khan, K. A. & Asiri, A. M. (2011). *J. Fluorine Chem.* **132**, 131–137.

[bb5] Faidallah, H. M. & Makki, M. S. I. (1994). *J. Chin. Chem. Soc.* **41**, 585–589.

[bb6] Farrugia, L. J. (1997). *J. Appl. Cryst.* **30**, 565.

[bb7] Sheldrick, G. M. (2008). *Acta Cryst.* A**64**, 112–122. 10.1107/S0108767307043930 18156677

[bb8] Westrip, S. P. (2010). *J. Appl. Cryst.* **43**, 920–925.

